# A time-resolved proteotranscriptomics atlas of the human placenta reveals pan-cancer immunomodulators

**DOI:** 10.1038/s41392-020-00224-5

**Published:** 2020-06-30

**Authors:** Na Ding, Botao Zhang, Wantao Ying, Jie Song, Lin Feng, Kaitai Zhang, Hongxia Li, Juan Xu, Ting Xiao, Shujun Cheng

**Affiliations:** 1 College of Bioinformatics Science and Technology, Harbin Medical University, Harbin, 150081 China; 2 State Key Laboratory of Molecular Oncology, Department of Etiology and Carcinogenesis, National Cancer Center/National Clinical Research Center for Cancer/Cancer Hospital, Chinese Academy of Medical Sciences and Peking Union Medical College, Beijing, 100021 China; 3 Department of Neuro-oncology, Neurosurgery Center, Beijing Tiantan Hospital, Capital Medical University, Beijing, 100070 China; 4 State Key Laboratory of Proteomics, Beijing Proteome Research Center, National Center for Protein Sciences (Beijing), Beijing Institute of Lifeomics, Beijing, 102206 China; 5 Department of Obstetrics and Gynecology, Beijing Shijitan Hospital, Capital Medical University, Beijing, 100038 China

**Keywords:** Computational biology and bioinformatics, Cancer

**Dear Editor**,

The placenta separates fetus and mother, with the trophoblast playing a most important role in defense against potential harm from the maternal immune system. Although the placenta is normal tissue, it shares several common features with malignant cells.^1^ Based on similar strategies, these cells are able to successfully coexist in an immunologically hostile environment.^2^ A detailed analysis and comparison of different placental model systems will not only contribute to our in-depth understanding of the exciting field of placental development research but also may offer valuable insights on the broad field of cancer studies.

The transcriptome profiling of human placenta has led to the landmark discovery of placentation and novel biomarkers for therapy response.^3^ However, the majority of these studies on placental development solely based on gene expression and without knowledge of related changes in the proteome are insufficient. Because proteins are the ultimate functional drivers of biology, are targets for drug actions and constitute potential biomarkers. Particularly, proteins appear to be important components in regulating the signal transduction required for immunological reactions. Therefore, an integrated study on the placental proteome and transcriptome, here termed proteotranscriptomics, in immune regulation will be essential to identify the immunomodulators in placental development and cancer.

In this study, 15 immature placental tissues and 6 mature placental tissues were sequenced (Supplementary Fig. S1a). The use of human tissue samples and experimental procedures for this study were reviewed and approved by the Ethics Committee of the Cancer Institute and Hospital, Chinese Academy of Medical Sciences. Liquid chromatography-tandem mass spectrometry (LC-MS/MS) was performed for the proteomic profiles, and mRNA transcriptome profiles of the corresponding samples assayed were constructed by RNA-seq. The flowchart of this study is depicted in Fig. 1a. Finally, we selected 6494 proteins and 12,924 mRNAs with accurate quantification for subsequent analysis. Among these, most proteins were shared by placental development, only a fraction was developmental stage specific (Supplementary Fig. S1b). Functional enrichment analysis indicated that specific expressed proteins in immature placenta were primarily involved in DNA repair, cell cycle, and regulation of lymphocyte activation and, etc. (Supplementary Fig. S1c). In almost every organism that has been examined to date, steady-state transcript abundances only partially predict protein abundance.^4^ The matched LC-MS/MS and RNA-seq measurements allowed the first global analysis of protein-mRNA relationships in the human placenta cohort. Consistent with previous findings, the correlation of mRNA and protein in this study was also moderate (median Spearman correlation coefficients (SCCs) = 0.44). Genes with positive correlations were mainly enriched in pathways including focal adhesion and the ECM receptor, genes with negative correlations were enriched in the spliceosome process and ribosomes, suggesting that the expression tendency has biological function effect (Fig. 1b).

**Fig. 1:**
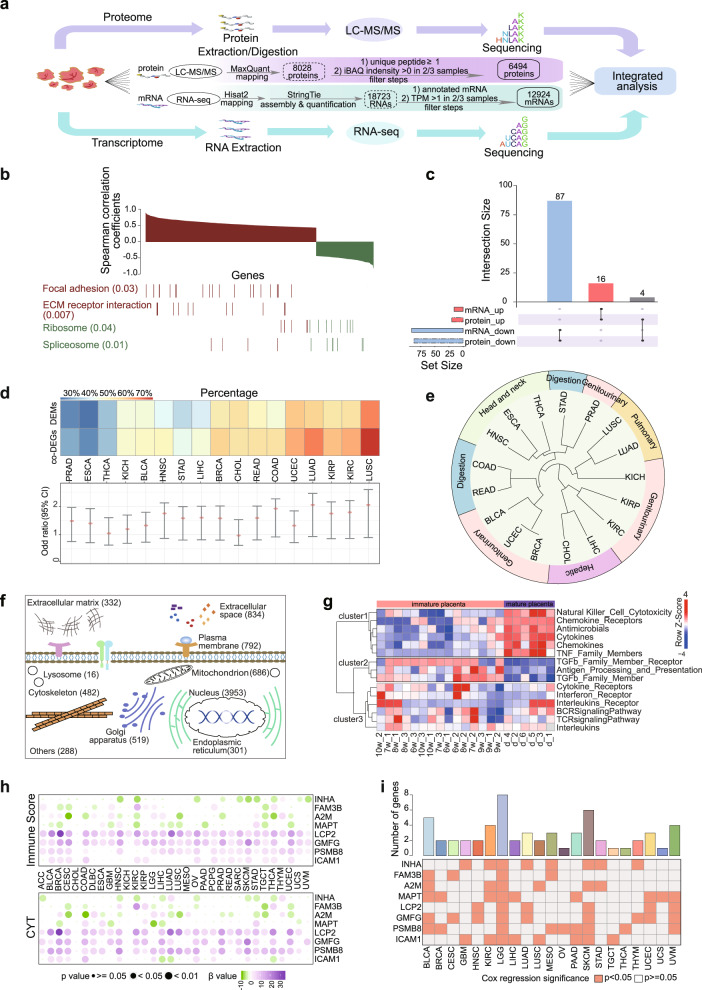
**a** Overview of the proteotranscriptomics atlas of human placenta samples. **b** The Spearman correlation coefficients correlation across each gene product. Positively correlated genes and their enriched pathways are shown in red, and negatively correlated genes and their enriched pathways are shown in green. Multiple-test adjusted *p* values are provided in the parentheses following the KEGG pathway names. **c** The number of common DEPs and DEMs. Each column represents the number of proteins intersecting with corresponding mRNAs. **d** The upper heatmap shows the proportion of placental DEM and co-DEGs. The lower panel shows the odds ratios and 95% confidence levels of Fisher’s exact test in each cancer type. **e** Clustering of cancer types with similar tissue origins based on the placental co-DEGs. **f** Subcellular location of placental expressed proteins, the numbers in brackets represent the number of proteins corresponding to the subcellular localization. **g** ssGSEA for placental proteome profiles and immune-related pathways. **h** The bubble chart displays the results of regression analysis of the key immunomodulators and cancer immunity scores (upper) and CYT (lower). Each column represents one cancer type, and each row represents a key immunomodulator. **i** Upper, the number of immunomodulators significantly associated with the patient prognosis across cancer types. And below heatmap represents the Cox regression significance of each key immunomodulators

Importantly, we investigated the landscape of protein/mRNA perturbations during placental development, fold change >2 or <0.5 and adjusted *P* value < 0.05 were considered as significant. In total, 103 co-differentially expressed genes with the same perturbation direction were selected as target genes (Fig. 1c), herein, called co-DEGs (Supplementary Table S1). To further screen for cancer genes, placental co-DEGs and placental differentially expressed mRNAs (DEMs) were compared in TCGA cancer samples. We found that placental co-DEGs were more likely to show expression perturbations than the placental DEMs across cancer types (Fig. 1d). Of the majority of co-DEGs from developing placenta were annotated as cancer hallmarks genes according to the annotations in MSigDB (Supplementary Fig. S2a). Interestingly, cancer types with similar tissue origins exhibited similar co-DEGs dysregulation patterns (Fig. 1e). Furthermore, the number of cancer types in which the placental co-DEGs occurred was calculated. We found that the distribution of this number follows a nonuniform distribution (Supplementary Fig. S2b), indicating the existence of distinct types of co-DEGs. We thus divided the co-DEGs into three groups based on the number of cancer types detected: seldom co-DEGs, moderate co-DEGs and pan-cancer co-DEGs (Supplementary Fig. S2b). To gain insights into the recurrence of these co-DEGs, a Circos plot was constructed to illustrate the detailed transcriptional alterations across cancer types (Fig. S2c). In addition, we extracted the protein interaction network mediated by the co-DEGs (Supplementary Fig. S2d). The degree distributions of the network were examined, which exhibited a power law distribution (Supplementary Fig. S2e). Moreover, a higher degree and lower characteristic path length were observed for pan-cancer co-DEGs than other categories (Supplementary Figs. S2f, S2g), suggesting that pan-cancer co-DEGs were likely to play pivotal roles and interact with each other in tumorigenesis. Moreover, 15/40 of the pan-cancer co-DEGs were known pharmacologically active target genes according to the annotations in DrugBank (Supplementary Fig. S2d, Supplementary Table S1), indicating their broad therapeutic effects. Overall, the specific features of the placental co-DEGs further highlight their critical roles in cancer.

As shown in Fig. 1f, a large number of proteins were annotated as extracellular matrix or in the extracellular space by the Human Protein Atlas, suggesting that our proteome datasets include the placental microenvironment. Thus, we particularly focused on distinct immune pathways derived from ImmPort to investigate the changes in immune regulation during placenta maturation. Single sample gene set enrichment analysis (ssGSEA) results showed that 9 immune-related pathways included in two clusters were switched between the immature and mature placenta, such as Natural Killer_Cell_Cytotoxicity (included in cluster1) and TGFb_Family_Member (included in cluster 2) (Fig. 1g). Furthermore, we identified 8 key immunomodulators based on two conditions: immunomodulators that participate in pathway alterations and those are placental co-DEGs. In addition, we characterized the 8 key immunomodulators in TCGA solid tumors. These key immunomodulators tended to be differentially expressed not only among cancer types but also in the same of perturbation direction between placenta and tumor (Supplementary Fig. S3a). For example, INHA was significantly upregulated in 11 cancers, and A2M showed significantly decreased expression across 12 cancer types (Supplementary Fig. S3b). Furthermore, we investigated the incidence relationship between key immunomodulators and pan-cancer immune, including immune scores and immune cytolytic activity (CYT) scores. These scores have been demonstrated to be useful biomarkers for predicting immune response.^5^ We calculated interdependent quantitative relationships using linear regression and Spearman correlation analysis, all the results suggested that the key immunomodulators have a significant influence on the immune process in most cancer cases (Fig. 1h, Supplementary Fig. S3c). To further demonstrate this result, the Wilcoxon rank sum test was used to assess the perturbations of the immune-related scores between patients with different expression of key immunomodulators across cancer types. We found that the immune scores of patients with varied expression of key immunomodulators were significantly different (Supplementary Fig. S3d). Moreover, the Cox risk regression illustrated the utility of key immunomodulators in evaluating the survival times and survival states of tumor patients (Fig. 1i). Overall, the expression of these key immunomodulators greatly contribute to tumor immune events and tumor patients’ prognosis.

In summary, we systematically characterized proteotranscriptomics profiles collected from time-resolved human placental tissues. Our study emphasizes an additional layer of immune system complexity, and it provides valuable assets that enable further data examination to obtain clues for a better understanding of placental development and tumorigenesis. The continued investigation of immune-related genes identified herein provide novel targets for diagnosis and treatment, and the finding offer insights into better clinical applications for both pathological pregnancies and human cancers.

## Supplementary information

Supplementary information

Supplementary information

Supplementary Table

## Data Availability

The raw proteome data have been submitted to iProX (accession number: IPX0001729000). The raw transcriptome data have been submitted to Genome Sequence Archive (accession number: HRA000050).
