# Supplementary material for: A time-resolved proteotranscriptomics atlas of the human placenta reveals pan-cancer immunomodulators
Source: Signal Transduct Target Ther. 2020 Jun 30;5:110. doi: 10.1038/s41392-020-00224-5 (PMC7327038; doi:10.1038/s41392-020-00224-5)
Supplement: Supplementary file 1 — Supplementary information [file 41392_2020_224_MOESM1_ESM.docx]

Supplementary Materials for

**A time-resolved proteotranscriptomics atlas of the human placenta reveals pan-cancer immunomodulators**

Na Ding^1,*^, Botao Zhang^2,4,*^, Wantao Ying^3,*^, Jie Song^2^, Lin Feng^2^, Kaitai Zhang^2^, Hongxia Li^5^, Juan Xu^1,#^, Ting Xiao^2,#^ and Shujun Cheng^2,1,#^

^1^College of Bioinformatics Science and Technology, Harbin Medical University, Harbin 150081, China;

^2^State Key Laboratory of Molecular Oncology, Department of Etiology and Carcinogenesis, National Cancer Center/National Clinical Research Center for Cancer/Cancer Hospital, Chinese Academy of Medical Sciences and Peking Union Medical College, Beijing, 100021, China;

^3^State Key Laboratory of Proteomics, Beijing Proteome Research Center, National Center for Protein Sciences (Beijing), Beijing Institute of Lifeomics, Beijing, 102206, China;

^4^Department of Neuro-oncology, Neurosurgery Center, Beijing Tiantan Hospital, Capital Medical University, Beijing, 100070, China;

^5^Department of Obstetrics and Gynecology, Beijing Shijitan Hospital, Capital Medical University, Beijing, 100038, China.*These authors contributed equally to this work.

Correspondence e-mail: chengshj@cicams.ac.cn to Shujun Cheng

xiaot@cicams.ac.cn to Ting Xiao

xujuanbiocc@ems.hrbmu.edu.cn to Juan Xu

**This file includes:**

Materials and Methods

Figures S1-S3

**Materials and Methods**

Sample selection and preparation

The human placental samples were obtained from Beijing Shijitan Hospital between March 2015 and August 2016. The enrolled samples were immature placental tissues from legal, elective pregnancy terminations by curettage and mature fetal placenta after term births. Six sampling time points were included, hereafter referred to as “6w”, “7w”, “8w”, “9w”, “10w” and “delivery” (detailed information regarding the number of samples is presented in Fig. 1a). For the abortion samples, women with a history of spontaneous abortion or developmental arrest history were excluded from this study. In the case of full-term delivery, women with pregnancy complications such as preeclampsia, fetal growth restriction, gestational diabetes and fetuses with known or suspected genetic diseases were also excluded from the study. Gestational age is based on the first day of the last menstrual period.

The tissue samples for the proteomic analysis were first washed by phosphate-buffered saline (PBS) and frozen in liquid nitrogen for storage before use. The tissue samples for RNA-seq were placed in RNAlater RNA Stabilization Reagent (Ambion/Thermo Fisher, Waltham, MA, USA) at 4°C overnight and then stored at -80°C until use.

Protein extraction and LC-MS/MS analysis

The tissues were lysed in a buffer consisting of 500 μL of 8 M urea in 0.1 M Tris/HCl with pH 8.5 (Invitrogen, USA) (UA), and 5 μL of a protease inhibitor cocktail (Applygen Technologies Inc., Beijing, China) on ice for 30 min. The lysate was sonicated for 10 min and centrifuged at 16,000 g at 4°C for 10 min. The proteins were digested with trypsin according to the filter-aided sample preparation protocol. In all, 200 μg of total protein was added, and the samples were centrifuged at 14,000 g for 10 min. Then, filters were washed twice with 200 μL of 8 M UA. Then 200 μL of 8 M urea and 2 μL of 1 M DTT were added to the filters and the samples were incubated at 37°C for 4 h. Samples were centrifuged at 14,000 g for 15 min. Then, 5 μL of 1 M iodoacetamide in 200 μL of 8 M urea were added to the filters, and the samples were incubated in darkness for 30 min. The filters were washed twice with 200 μL of 8 M UA, followed by two washes with 200 μL of 50 mM NH_4_HCO_3_. Finally, trypsin was added in 200 μL of 50 mM NH_4_HCO_3_ to each filter. The protein-to-enzyme ratio was 50:1. The samples were incubated overnight at 37°C, and peptides were collected by centrifugation.

The LC-MS/MS detection system consisted of a nanoflow high-performance liquid chromatography (HPLC) instrument (UltiMate 3000, Thermo Fisher Scientific, MA, USA) coupled to a Q-Exactive HF mass spectrometer (MS) (Thermo Fisher) with a nanoelectrospray ion source (Thermo Fisher). In brief, 0.5 μg of peptide mixture resolved in buffer A (0.1% formic acid (FA)) was loaded onto a 2-cm self-packed trap column (100-μm inner diameter, ReproSil-Pur C18-AQ, 3 μm; Dr. Maisch) using buffer A and separated on a 75-μm inner-diameter column with a length of 12 cm (ReproSil-Pur C18-AQ, 3 μm; Dr. Maisch) over a 78-min gradient (buffer A, 0.1% FA in water; buffer B, 80% ACN/ 0.1% FA in water) at a flow rate of 600 nL/min (B%: 0 min, 6%; 8 min, 9%; 24 min, 14%; 60 min, 30% B; 75 min, 40% B; 78 min, 95%). For the full MS survey scan, the target value was 3 × 10^6^ and the scan ranged from 300 to 1400 m/z at a resolution of 120,000 and a maximum injection time of 80 ms. For the MS2 scan, the top 20 mode was adopted. Only spectra with a charge state of 2–6 were selected for fragmentation by higher-energy collision dissociation with a normalized collision energy of 27%. The MS2 spectra were acquired in the Orbitrap with an AGC target of 5 × 10^4^ and a maximum injection time of 45 ms.

Identification and quantification of proteins

The tandem mass spectra were searched against the human UniProt database (version 20140922; 20,193 sequences) using MaxQuant (version 1.5.3.30). Trypsin was selected as the proteolytic enzyme, and two missed cleavage sites were allowed. Cysteine carbamidomethylation was set as the fixed modification. The oxidation of M and acetylation of the protein N-terminal were set as the variable modifications. The first search mass tolerance was 20 ppm, and the main search peptide tolerance was 4.5 ppm. The FDR of the peptide-spectrum matches (PSMs) and proteins were set to less than 1%.

For the quantitative analysis of the proteomics data, the iBAQ intensities of the samples were extracted from the MaxQuant result files to represent the final expression of a particular protein across samples. Then, the expression matrix was subjected to quantile normalization using normalized quantile functions implemented in the R/Bioconductor package “limma” (version 3.34.9), which is an optimal normalization method commonly used in shotgun proteomics data analysis. Missing values were imputed with the minimum value across our proteomic data.

RNA extraction and RNA-seq

Total RNA was isolated from frozen chorionic villus and mature placental tissues with TRIzol reagent (Thermo Fisher, USA) according to the manufacturer’s instructions. A complementary DNA library was prepared, and sequencing was performed according to the Illumina standard protocol by Beijing Novel Bioinformatics Co., Ltd. (https://en.novogene.com/). Raw reads from the RNA-seq libraries were trimmed to remove the adaptor sequence, reads with adaptor contaminants and low-quality reads.

Identification and quantification of mRNAs

The RNA-seq reads per sample were mapped to the reference using HISAT2 (version 2.0.5) with default parameters. The reference consisted of the human reference genome and the Ensembl annotated human transcriptome (GRCh38/hg38 assembly). Then, the mapped reads were processed using StringTie (version 1.3.3). For the quantitative analysis of the transcriptome data, the TPM were used for mRNA expression.

Evaluation of the expression correlation at the protein and mRNA levels

The protein-mRNA correspondence was obtained from the UniProt database. Among 6494 proteins, only 6060 genes were included in the RNA-seq data set. For each of the 21 samples, we calculated the Spearman correlation coefficient (SCC) between iBAQ intensities for protein and TPM for RNA of 6060 genes. Next, to compare protein and mRNA across samples, we calculated the SCC between iBAQ intensities and the TPM of each gene. Then, p values corresponding to the coefficients were computed and adjusted by the Benjamini–Hochberg procedure. Significance was established based on an adjusted p value cutoff of 0.05.

Identification of DEPs, DEMs and co-DEGs

To identify the differentially expressed proteins (DEPs), the statistical significance was calculated by a simple linear model and moderated t-statistics using an empirical Bayes shrinkage method implemented in the R package “limma”. Fold changes and Benjamini–Hochberg-adjusted p values were extracted from all comparison results. The R package “pheatmap” was used for cluster analysis.

The method for identifying differentially expressed mRNAs (DEMs) was similar to that for DEPs.

We selected co-DEG based on two condition: (1) The gene was significantly differentially expressed at both transcriptome and proteome levels; (2) The direction of differential expression was consistent at two levels. For example, a co-DEG should be upregulated at both the protein and mRNA levels, and vice versa.

The mRNA transcriptome landscape across tumor types

RNA-Seq based gene expression profile data were obtained from the Cancer Genome Atlas (TCGA) project via the R package “TCGAbiolinks”. We downloaded the Fragments Per Kilobase of transcript per Million mapped reads (FPKM)-based gene expression for 32 types of solid tumors. Based on the gene annotations in Ensembl, we extracted the protein-coding gene expression profiles for each cancer type. The clinical information of the cancer patients was also downloaded from the TCGA project, including the survival status and survival. Here, we considered cancer types with more than five normal samples, and mRNAs with an expression level of 0 in more than 30% of the samples were excluded in subsequent analysis. We identified DEMs in each cancer type by t-tests. The mRNAs with fold changes >2 or <1/2 and p value <0.05 were identified as differentially expressed in each cancer type.

Immune-related scores of cancer patients

We calculated two immune response-related scores that were estimated from gene expression. Immune scores that represent the infiltration of immune cells in tumor tissues were estimated based on the R package “ESTIMATE”. In addition, we used a quantitative measure of immune cytolytic activity (CYT) based on the expression levels of granzyme A (GZMA) and perforin (PRF1)

The correlation analysis between the expression of key immunomodulators and the immune-related scores of cancer patients

The SCCs between expression of key immunomodulators and immune response-related scores in each cancer types were calculated. We separately counted the number of cancers in which the immunomodulators were significantly correlated with immune scores and CYT levels. Significance was established based on p value cutoff of 0.05. To further demonstrate this result, we sorted the expression of each key immunomodulators from high to low in each cancer type, and divided the top 30% and bottom 30% into two groups. The Wilcoxon rank sum test was performed to assess the differences of immune-related scores between the two groups. The p value < 0.05 was considered significant.

**DATA AVAILABILITY**

The raw proteome data have been submitted to iProX (accession number: IPX0001729000). The raw transcriptome data have been submitted to Genome Sequence Archive (accession number: HRA000050).

**Figure. S1**


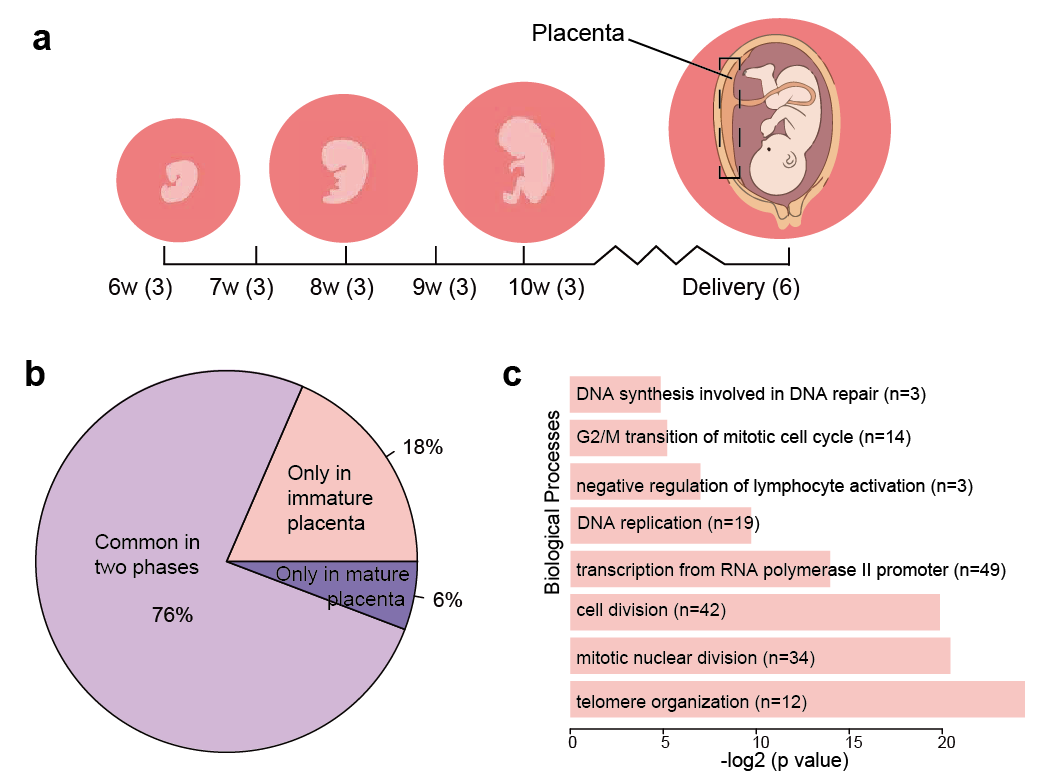


**Fig. S1 Proteotranscriptomic atlas of the human placenta. a** Diagrammatic sample information. The location of the sample selection is marked by a dotted box. The bottom characters are sampling time points, and the numbers in parentheses represent the number of samples. **b** The pie chart shows the proportion of genes that express both protein and mRNA detected in different placental development stages. **c** Biological processes enriched by immature placenta-specific proteins. The numbers in brackets represent the number of proteins involved in the function term.

**Figure. S2**


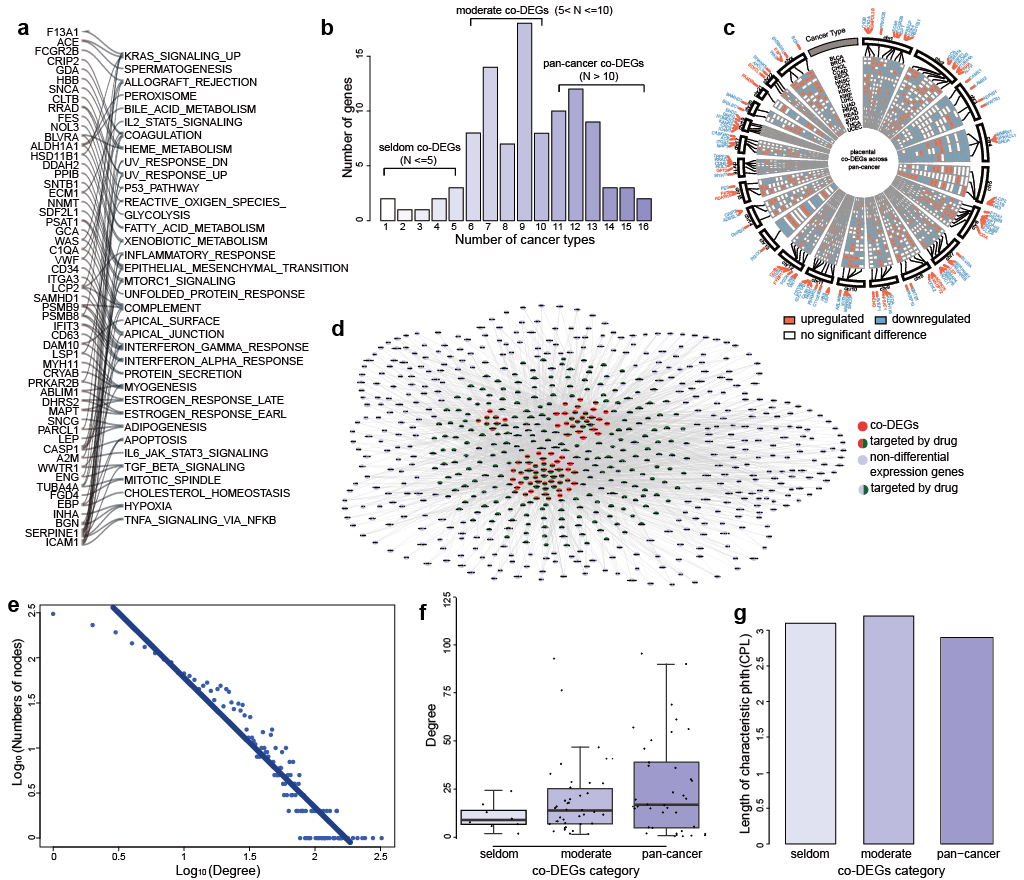


**Fig. S2 Characterization of cancer genes utilizing placental co-DEGs. a** The riverplot shows that the placental co-DEGs are connected to cancer hallmarks**. b** The number of co-DEGs that occur in different cancer types. co-DEGs are classified into three types: cancer seldom (occurring in less than 5 cancer types), moderate (occurring in 5–10 cancer types) and pan-cancer (occurring in more than 10 cancer types). **c** The Circos plot represents the different states of 103 co-DEGs in each cancer type. Red and blue colors represent upregulated and downregulated separately, blank represents no significant difference in expression. **d** PPI network mediated by placental co-DEGs, which was drawn by Cytoscape (revision 3.6.1). The node categories are shown in the top right legend. **e** The degree distribution of placental co-DEGs-mediated PPI network. **f** The degree distribution of three types of co-DEG nodes. In the box, the middle bar represents the median, and the box represents the interquartile range; error bars extend to 1.5× the interquartile range; each dot indicates the number of proteins to which the node is connected. **g** The CPL of three kinds of co-DEGs.

**Figure. S3**


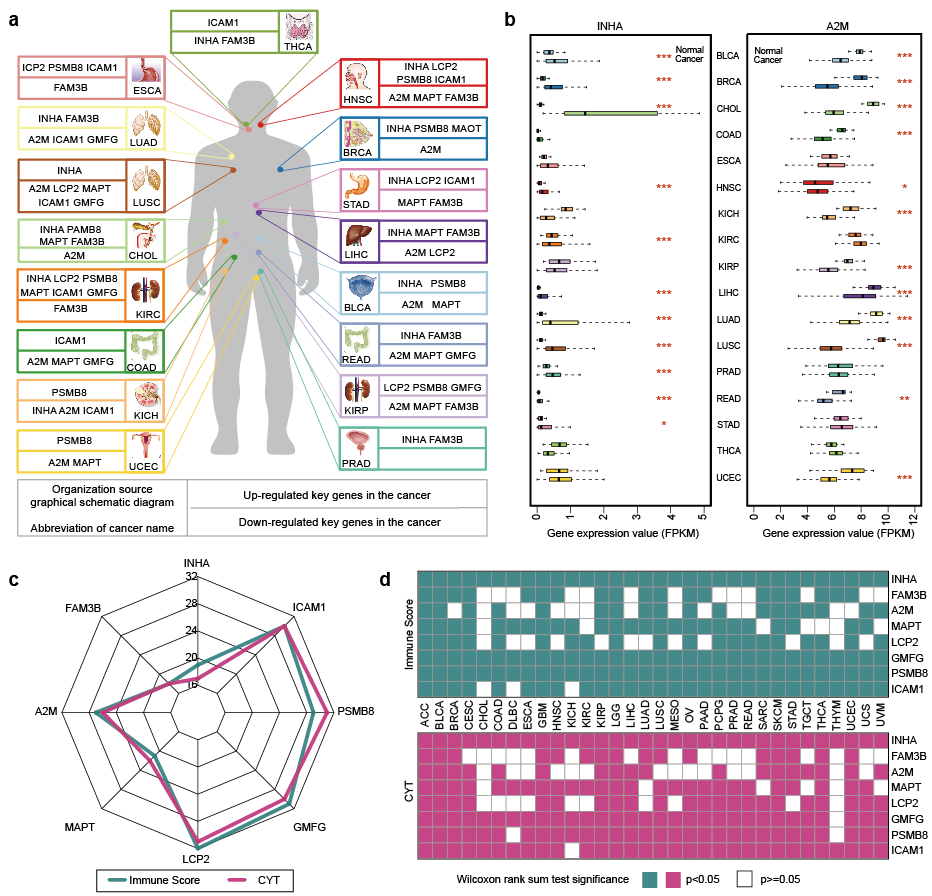


**Fig. S3 Key immunomodulators across cancer types. a** The graphics and text describe the upregulated and downregulated key immunomodulators across 32 solid tumors. **b** The expression of INHA and A2M in normal and cancer samples across cancer types. In the box, the middle bar represents the median, and the box represents the interquartile range; error bars extend to 1.5× the interquartile range. ***P < 0.001, **p<0.01 and *P < 0.05, Student's t test. **c** The number of cancers in which the key immunomodulators were significantly correlated with immune scores and CYT levels. The grid lines represent the number of cancer types. **d** The heatmap represents the Wilcoxon rank sum test significance of the immune-related score difference between patients with upper 30% and lower 30% expression of each key immunomodulators across cancer types.
