# Supplementary material for: A time-resolved proteotranscriptomics atlas of the human placenta reveals pan-cancer immunomodulators
Source: Signal Transduct Target Ther. 2020 Jun 30;5:110. doi: 10.1038/s41392-020-00224-5 (PMC7327038; doi:10.1038/s41392-020-00224-5)
Supplement: Supplementary file 3 — Supplementary Table [file 41392_2020_224_MOESM3_ESM.pdf]

Uniprot.ID	Gene.symbol	Dif_placenta_Label	Cancer_Label	Drug.ID
O14672	ADAM10	up	seldom	DB04991
O95865	DDAH2	down	seldom	DB00155
P12821	ACE	down	seldom	DB00178; DB00492; DB00519; DB00542; DB00584; DB00616; DB00691; DB00722; DB00790; DB00881; DB00886; DB01180; DB01197; DB01340; DB01348; DB02032; DB03740; DB08836; DB09477; DB13166
P28676	GCA	down	seldom	DB11093; DB11348; DB14481
AGPAT5	Q9NUQ2	up	seldom	
CASKIN2	Q8WXE0	down	seldom	
CGA	P01215	up	seldom	
CLTB	P09497	down	seldom	
LEP	P41159	up	seldom	
O14791	APOL1	down	pan-cancer	DB01593; DB14487; DB14533
P00352	ALDH1A1	down	pan-cancer	DB00157; DB00162; DB00755; DB04447
P00488	F13A1	down	pan-cancer	DB01839; DB02340; DB11300; DB11311; DB11571; DB11572; DB13151
P01023	A2M	down	pan-cancer	DB00102; DB00515; DB00626; DB01593; DB08888; DB12965; DB14487; DB14533
P02746	C1QB	down	pan-cancer	DB00002; DB00005; DB00054; DB00056; DB00072; DB00074; DB00075; DB00078; DB00081; DB00087; DB00092; DB00095; DB00108; DB00110; DB00111; DB00112; DB01593; DB14487; DB14533
P10636	MAPT	down	pan-cancer	DB00448; DB00637; DB01229; DB01248
P19801	AOC1	down	pan-cancer	DB00594; DB01373; DB03608
P31323	PRKAR2B	down	pan-cancer	DB02527
P35749	MYH11	down	pan-cancer	DB04444
P37840	SNCA	down	pan-cancer	DB02709; DB09130
P68871	HBB	down	pan-cancer	DB00893; DB01593; DB02126; DB06154; DB07427; DB07428; DB07645; DB08077; DB08262; DB08486; DB08632; DB09112; DB09130; DB09140; DB09147; DB09517; DB13995; DB14487; DB14533
Q13938	CAPS	down	pan-cancer	DB11093; DB11348; DB14481
Q8TD30	GPT2	up	pan-cancer	DB00114; DB00142; DB00160; DB00780
Q9BZZ2	SIGLEC1	down	pan-cancer	DB02379; DB03721
Q9Y617	PSAT1	up	pan-cancer	DB00114; DB00142
ABLIM1	O14639	down	pan-cancer	

BGN	P21810	down	pan-cancer	
CD34	P28906	down	pan-cancer	
CLIC5	Q9NZA1	down	pan-cancer	
CRYAB	P02511	down	pan-cancer	
DPT	Q07507	down	pan-cancer	
ECM1	Q16610	down	pan-cancer	
EHD2	Q9NZN4	down	pan-cancer	
FAM3B	P58499	up	pan-cancer	
GIMAP8	Q8ND71	down	pan-cancer	
GRB14	Q14449	down	pan-cancer	
HK3	P52790	down	pan-cancer	
HOMER3	Q9NSC5	down	pan-cancer	
INHA	P05111	up	pan-cancer	
MMRN1	Q13201	down	pan-cancer	
PRELP	P51888	down	pan-cancer	
SDF2L1	Q9HCN8	up	pan-cancer	
SH3BGRL	O75368	down	pan-cancer	
SMPDL3B	Q92485	up	pan-cancer	
SNCG	O76070	down	pan-cancer	
SPARCL1	Q14515	down	pan-cancer	
SVEP1	Q4LDE5	down	pan-cancer	
TNS2	Q63HR2	down	pan-cancer	
WWTR1	Q9GZV5	down	pan-cancer	
LVRN	Q6Q4G3	down	pan-cancer	
P02745	C1QA	down	moderate	DB00002; DB00005; DB00054; DB00056; DB00072; DB00074; DB00075; DB00078; DB00081; DB00087; DB00092; DB00095; DB00108; DB00110; DB00111; DB00112
P04275	VWF	down	moderate	DB00025; DB05202; DB06081; DB09108; DB09329; DB11606; DB11607; DB13998; DB13999
P05121	SERPINE1	down	moderate	DB00009; DB00013; DB00015; DB00029; DB00031; DB00055; DB00197; DB05254; DB09130
P05187	ALPP	down	moderate	DB01373; DB08413
P05362	ICAM1	down	moderate	DB00108; DB08818; DB12598
P07332	FES	down	moderate	DB12010
P22694	PRKACB	down	moderate	DB02482; DB12010
P23284	PPIB	up	moderate	DB00172; DB04447
P28062	PSMB8	down	moderate	DB08889
P28065	PSMB9	down	moderate	DB08889
P28845	HSD11B1	down	moderate	DB00157; DB00635; DB02329; DB03461; DB03814; DB04652; DB05064; DB06992; DB07017; DB07049; DB07056; DB07310; DB07316; DB07619; DB07624; DB07866; DB08277; DB08280; DB08771; DB13751

P29466	CASP1	down	moderate	DB01017; DB04875; DB05301; DB05408; DB05507; DB07733; DB07744; DB07916
P31994	FCGR2B	down	moderate	DB00002; DB00005; DB00028; DB00054; DB00056; DB00072; DB00074; DB00075; DB00078; DB00081; DB00087; DB00092; DB00095; DB00098; DB00108; DB00110; DB00111; DB00112; DB11767
P33151	CDH5	down	moderate	DB00480; DB05685
P33402	GUCY1A2	down	moderate	DB00435; DB01020; DB08931; DB09241; DB09282; DB13170
P40261	NNMT	down	moderate	DB00627
P42768	WAS	down	moderate	DB01731
P53004	BLVRA	down	moderate	DB00157
P68366	TUBA4A	down	moderate	DB00541; DB01179; DB01873; DB03010; DB05147; DB05281; DB06772; DB12695
Q14012	CAMK1	down	moderate	DB12010
Q15125	EBP	up	moderate	DB00675
Q8N142	ADSSL1	down	moderate	DB00128; DB02109; DB03510; DB04137; DB04315; DB04418; DB04566; DB05540
ADPRH	P54922	down	moderate	
AIF1	P55008	down	moderate	
ANGPT2	O15123	up	moderate	
C11orf96	Q7Z7L8	down	moderate	
CD63	P08962	up	moderate	
CRIP2	P52943	down	moderate	
DHRS2	Q13268	down	moderate	
DOK2	O60496	down	moderate	
ENG	P17813	up	moderate	
FERMT3	Q86UX7	down	moderate	
FGD4	Q96M96	down	moderate	
FKBP11	Q9NYL4	up	moderate	
GDA	Q9Y2T3	down	moderate	
GIMAP4	Q9NUV9	down	moderate	
GMFG	O60234	down	moderate	
HECW2	Q9P2P5	down	moderate	
IFIT3	O14879	down	moderate	
INPP1	P49441	down	moderate	
ITGA3	P26006	down	moderate	
KANK3	Q6NY19	down	moderate	
LAMB2	P55268	down	moderate	
LCP2	Q13094	down	moderate	
LSP1	P33241	down	moderate	
MMRN2	Q9H8L6	down	moderate	
NOL3	O60936	down	moderate	
PARP10	Q53GL7	down	moderate	

RGCC	Q9H4X1	down	moderate
RRAD	P55042	down	moderate
SAMHD1	Q9Y3Z3	down	moderate
SHANK3	Q9BYB0	down	moderate
SNTB1	Q13884	down	moderate
TPPP3	Q9BW30	down	moderate
